# Abscess of Hip

**Published:** 1881-04

**Authors:** 


III.—Abscess of Hip.—On February 22d a horse was brought to the hospital for lameness of the right posterior extremity, with the history that he had bruised the ham of that side in pushing through a narrow alley-way. On examination, a large swelling was noticed over the right acetabulum, extending over considerable territory. Upon palpation, fluctuation was detected ; the tumor was aspirated, and found to contain pus, and an abscess was diagnosticated. An incision was made into the abscess two inches behind the trochanter of the femur, which seemed to be the most dependent portion. There was a free discharge of pus and a rapid disappearance of the swelling. A probe was introduced into the cavity, and it was found to have extended nearly up to the most prominent portion of the hip. Free drainage was established by two counter openings and the introduction of setons. The wounds were carefully cleansed by carbolic acid solution (1 to 40). The abscess healed rapidly, and on March 8th the animal was discharged, cured.

